# Re-appraisal of the obesity paradox in heart failure: a meta-analysis of individual data

**DOI:** 10.1007/s00392-021-01822-1

**Published:** 2021-03-11

**Authors:** Nick Marcks, Alberto Aimo, James L. Januzzi, Giuseppe Vergaro, Aldo Clerico, Roberto Latini, Jennifer Meessen, Inder S. Anand, Jay N. Cohn, Jørgen Gravning, Thor Ueland, Antoni Bayes-Genis, Josep Lupón, Rudolf A. de Boer, Akiomi Yoshihisa, Yasuchika Takeishi, Michael Egstrup, Ida Gustafsson, Hanna K. Gaggin, Kai M. Eggers, Kurt Huber, Ioannis Tentzeris, Andrea Ripoli, Claudio Passino, Sandra Sanders-van Wijk, Michele Emdin, Hans-Peter Brunner-La Rocca

**Affiliations:** 1 Department of Cardiology, Maastricht University Medical Centre, PO Box 5800, 6202AZ Maastricht, The Netherlands; 2 Cardiology Division, University Hospital of Pisa, Pisa, Italy; 3 Massachusetts General Hospital and Baim Institute for Clinical Research, Boston, USA; 4 Institute of Life Sciences, Scuola Superiore Sant’Anna, Pisa, Italy; 5 Fondazione Toscana G. Monasterio, Pisa, Italy; 6 Department of Cardiovascular Medicine, Institute for Pharmacological Research Mario Negri IRCCS, Milan, Italy; 7 Division of Cardiovascular Medicine, University of Minnesota, Minneapolis, USA; 8 Department of Cardiology, VA Medical Centre, Minneapolis, USA; 9 Department of Cardiology, Oslo University Hospital, Ullevål, Oslo, Norway; 10 Centre for Heart Failure Research, University of Oslo, Oslo, Norway; 11 Research Institute of Internal Medicine, Oslo University Hospital, Rikshospitalet, Oslo, Norway; 12 Faculty of Medicine, University of Oslo, Oslo, Norway; 13 K. G. Jebsen Thrombosis Research and Expertise Centre, University of Tromsø, Tromsø, Norway; 14 Hospital Universitari Germans Trias I Pujol, Badalona (Barcelona), Spain; 15 University Medical Centre Groningen, University Medical Centre Groningen, Groningen, The Netherlands; 16 Department of Cardiovascular Medicine, Fukushima Medical University, Fukushima, Japan; 17 Department of Cardiology, Bispebjerg University Hospital, København, Denmark; 18 Department of Medical Sciences, Cardiology, Uppsala University, Uppsala, Sweden; 19 Faculty of Internal Medicine, Wilhelminenspital and Sigmund Freud University Medical School, Vienna, Austria

**Keywords:** Heart failure, Obesity, Body mass index, Disease severity, Co-morbidities, Biomarkers

## Abstract

**Background:**

Higher body mass index (BMI) is associated with better outcome compared with normal weight in patients with HF and other chronic diseases. It remains uncertain whether the apparent protective role of obesity relates to the absence of comorbidities. Therefore, we investigated the effect of BMI on outcome in younger patients without co-morbidities as compared to older patients with co-morbidities in a large heart failure (HF) population.

**Methods:**

In an individual patient data analysis from pooled cohorts, 5,819 patients with chronic HF and data available on BMI, co-morbidities and outcome were analysed. Patients were divided into four groups based on BMI (i.e. ≤ 18.5 kg/m^2^, 18.5–25.0 kg/m^2^; 25.0–30.0 kg/m^2^; 30.0 kg/m^2^). Primary endpoints included all-cause mortality and HF hospitalization-free survival.

**Results:**

Mean age was 65 ± 12 years, with a majority of males (78%), ischaemic HF and HF with reduced ejection fraction. Frequency of all-cause mortality or HF hospitalization was significantly worse in the lowest two BMI groups as compared to the other two groups; however, this effect was only seen in patients older than 75 years or having at least one relevant co-morbidity, and not in younger patients with HF only. After including medications and N-terminal pro-B-type natriuretic peptide and high-sensitivity cardiac troponin concentrations into the model, the prognostic impact of BMI was largely absent even in the elderly group with co-morbidity.

**Conclusions:**

The present study suggests that obesity is a marker of less advanced disease, but does not have an independent protective effect in patients with chronic HF.

**Graphic abstract:**

Categories of BMI are only predictive of poor outcome in patients aged > 75 years or with at least one co-morbidity (bottom), but not in those aged < 75 years without co-morbidities (top). The prognostic effect largely disappears in multivariable analyses even for the former group. These findings question the protective effect of obesity in chronic heart failure (HF).

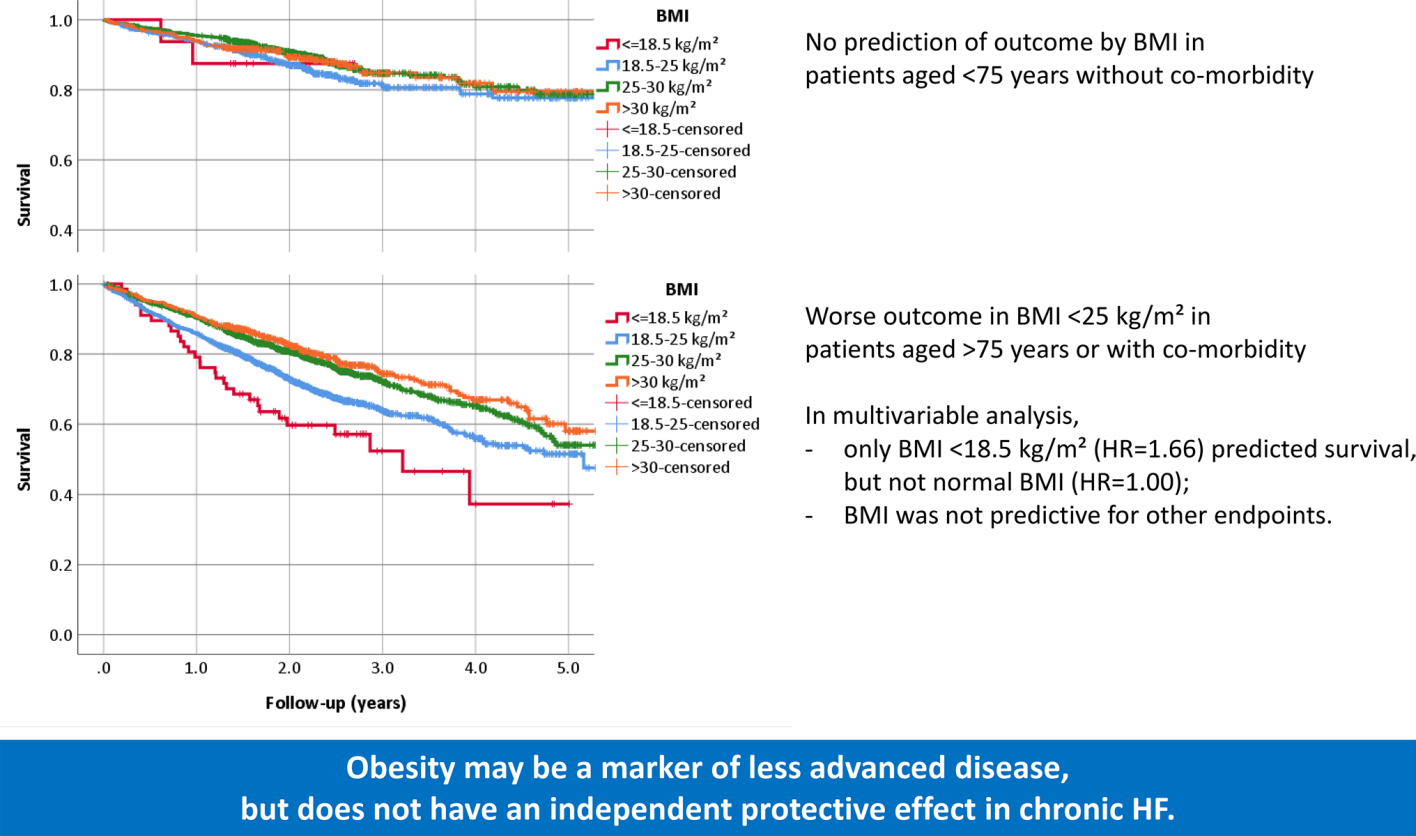

**Supplementary Information:**

The online version contains supplementary material available at 10.1007/s00392-021-01822-1.

## Introduction

Obesity is recognized as a major health care problem, increasing the risk of several cardiovascular diseases and other chronic disorders including heart failure (HF) [1]. However, in elderly patients and those with chronic diseases, overweight and mild-to-moderate obesity may be associated with better outcome compared to patients with normal weight. This association has also been found in patients with HF [2] and other cardiovascular diseases [3], where losing weight is associated with worse outcome [4]. The question arises if the latter is simply an expression of more advanced disease or has a direct negative cardiac impact. In fact, there is evidence that a catabolic state increases inflammation and might contribute to disease progression [5]. In addition, several factors associated with obesity may positively affect chronic diseases such as HF including an attenuated response to sympathetic and renin–angiotensin system activation, better tolerability of drugs for neurohormonal antagonism [6] and reduced peripheral vascular resistances by higher insulin concentrations [7]. In theory, however, these factors would also have beneficial effects in patients without chronic diseases, which obviously is not the case. Moreover, abdominal obesity might increase the risk of death in HF, at least among women [8]. Taken together, it remains uncertain if the so-called obesity paradox is simply related to the fact that older patients and those with more advanced disease(s) are unable to gain weight or often even lose weight, or if there is a direct protective effect of adipose tissue in chronic diseases. In a large dataset of individual HF patients, we, therefore, investigated the effect of body-mass index (BMI) as a measure of obesity on outcome in younger patients without co-morbidities as compared to older patients with co-morbidities.

## Methods

### Search strategy, study selection

In April 2017, studies evaluating high-sensitive cardiac troponin T (hs-cTnT) and prognosis in chronic HF were searched in four databases (Medline, EMBASE, Cochrane Library and Scopus). Investigators of the different studies were contacted to provide individual patient data to perform a meta-analysis on hs-cTnT and prognosis as previously reported [9] (see also supplementary material). For the present analysis, patients with data available on BMI, co-morbidities and outcome were considered (5819 out of 9289, 63%). As outcomes, all-cause mortality and HF hospitalization-free survival were assessed.

BMI was calculated as weight (kg)/height^2^ (m^2^). Patients were divided into four groups based on BMI (i.e. underweight [BMI ≤ 18.5 kg/m^2^]; normal [BMI > 18.5–25.0 kg/m^2^]; overweight [BMI > 25.0–30.0 kg/m^2^]; obese [BMI > 30.0 kg/m^2^]). In addition, data were analysed considering underweight (i.e. ≤ 18.5 kg/m^2^) and different classes of obesity (class 1: 30–35 kg/m^2^; class 2 35–40 kg/m^2^; class 3 > 40 kg/m^2^) separately.

Information of the following co-morbidities was available as defined in each individual study: hypertension, cancer, chronic obstructive pulmonary disease (COPD), diabetes, anaemia, and renal failure (defined as estimated glomerular filtration rate [eGFR] < 45 ml/min using the Chronic Kidney Disease Epidemiology collaboration equation). These co-morbidities were scored as either present or absent based on the medical history of patients. In addition, age, sex, ischaemic aetiology of HF, atrial fibrillation, systolic blood pressure, heart rate, New York Heart Association (NYHA)-class and the biomarkers N-terminal pro-B-type natriuretic peptide (NT-proBNP), high-sensitivity cardiac troponin T (hs-cTnT) and soluble suppression of tumorgenicity-2 (sST2) were considered. As sST2 was only known in a proportion of patients (*n* = 2191, 38%), sST2 was not used for the multivariable prediction model of outcome. The methods of analysing these biomarkers were reported previously [10].

Regarding the analysis of patients with and without co-morbidities, non-cardiovascular co-morbidities were considered, i.e. anaemia, COPD, diabetes, renal failure, and cancer. In addition, patients were separated based on their age, as elderly patients (> 75 years of age) very often have important co-morbidities even if they have not been specifically scored. Accordingly, patients were divided into two groups: one with none of the above mentioned non-cardiovascular co-morbidities and age below 75 years, and one with all other patients (i.e. either age > 75 years or the presence of at least one of the mentioned non-cardiovascular co-morbidities).

### Statistical analysis

Data are presented as mean ± standard deviation (SD) or as median and interquartile range (IQR), as appropriate. Discrete variables are reported as frequencies (percentage). Biomarker levels were log10-transformed for statistical comparisons. Between-group comparison was done using Chi-square (χ^2^), ANOVA or Kruskal–Wallis test, as appropriate. Kaplan–Meier curves were used to calculate (event-free) survival. log-rank testing was used to compare survival times. Nonlinear spline regression was used to determine the effect of BMI as continuous variable and outcome using R (V4.0). Cox-regression was used to calculate the hazard ratio (HR) in multivariable survival analysis. First, the BMI categories were entered into the equation and next the stepwise forward procedure was used using all variables with *p* < 0.1 in univariable analysis. The calculations were done separately in patients aged < 75 years with none of the above-mentioned non-cardiovascular co-morbidities and the other patients (i.e. > 75 years of age and/or the presence of at least one of the co-morbidities). Linear regression was used for the calculation of factors associated with log10-transformed biomarker levels. Again, BMI was first entered into the equation, followed by stepwise forward procedure for other variables. Using stepwise backward procedure did not change results for both Cox-regression and linear regression (data not shown). All calculations apart from spline regression were done using SPSS V26.0. A two-sided *p* value of < 0.05 was considered to be statistically significant.

## Results

### Baseline characteristics

Baseline characteristics of patients are shown in Table 1. They were on average 65 years old, the majority was male and the most important underlying cause of HF was coronary artery disease. Most patients had reduced left-ventricular ejection fraction (LVEF). Co-morbidities were common. Most patients were treated with angiotensin-converting-enzyme (ACE)-inhibitors or angiotensin-receptor blockers (ARB’s), but only about half received a β-blocker and a minority received treatment with a mineralocorticoid receptor antagonist (MRA). Biomarkers were moderately elevated.

**Table 1:** Baseline characteristics overall and in patients based on BMI groups

	All (*n* = 5819)	BMI < 18.5 (*n* = 83)	BMI 18.5–25 (*n* = 2062)	BMI 25–30 (*n* = 2503)	BMI ≥ 30 (*n* = 1171)	*p*
Age (years)	64.9 ± 11.5	69.1 ± 14.1	67.1 ± 11.7	64.8 ± 10.6	61.0 ± 11.6	< 0.001
Age ≥ 75 years	1053 (18.1%)	30 (36.1%)	517 (25.1%)	384 (15.3%)	122 (10.4%)	< 0.001
Male gender	4561 (78.4%)	36 (43.4%)	1563 (75.8%)	2067 (82.6%)	895 (76.4%)	< 0.001
Ischaemic aetiology	3256 (56.0%)	39 (47.0%)	1182 (57.3%)	1454 (58.1%)	581 (49.6%)	< 0.001
Hypertension	2776 (47.7%)	37 (44.6%)	841 (40.8%)	1231 (49.2%)	667 (57.0%)	< 0.001
Cancer	427 (7.3%)	8 (9.6%)	177 (8.6%)	167 (6.7%)	75 (6.4%)	0.04
COPD	860 (14.8%)	24 (28.9%)	295 (14.3%)	345 (13.8%)	196 (16.7%)	< 0.001
Diabetes	1563 (26.9%)	9 (10.8%)	446 (21.6%)	682 (27.2%)	426 (36.4%)	< 0.001
Haemoglobin (g/dl)	13.6 ± 1.5	12.8 ± 1.7	13.4 ± 1.5	13.7 ± 1.5	13.7 ± 1.5	< 0.001
Anaemia	1605 (27.6%)	32 (38.6%)	665 (32.3%)	634 (25.3%)	274 (23.4%)	< 0.001
eGFR	58.8 ± 17.2	58.5 ± 26.1	57.9 ± 17.6	58.5 ± 16.2	61.1 ± 17.7	< 0.001
eGFR < 45	1192 (20.5%)	30 (36.1%)	481 (23.3%)	489 (19.5%)	192 (16.4%)	< 0.001
Atrial fibrillation	893 (15.4%)	10 (12.0%)	305 (14.8%)	382 (15.3%)	196 (16.7%)	0.41
LVEF	28.9 ± 9.1	27.9 ± 10.6	28.3 ± 8.9	29.1 ± 8.6	29.8 ± 9.9	< 0.001
LVEF ≤ 40%	5527 (95.0%)	75 (90.4%)	1969 (95.5%)	2390 (95.5%)	1093 (93.3%)	0.004
NYHA III/IV	2282 (39.2%)	46 (55.4%)	879 (42.6%)	903 (36.1%)	454 (38.8%)	< 0.001
ACE/ARB	5242 (90.1%)	69 (83.1%)	1848 (89.6%)	2251 (90.0%)	1071 (91.7%)	0.04
β-Blocker	2706 (46.5%)	31 (37.3%)	921 (44.7%)	1192 (47.6%)	562 (48.0%)	0.05
MRA	954 (16.4%)	19 (22.9%)	359 (17.4%)	402 (16.1%)	174 (14.9%)	0.10
NT-proBNP (pg/ml)	1001 [416, 2345]	2587 [980, 5364]	1509 [613, 3303]	912 [392, 2020]	653 [277, 1409]	< 0.001
hs-cTnT (pg/ml)	14.8 [7.5, 26.3]	16.9 [9.4, 29.1]	15.9 [7.8, 29.5]	14.2 [7.4, 24.9]	14.7 [7.7, 24.0]	0.001
sST2 (ng/ml)	26.9 [20.2, 38.5]	31.2 [22.3, 39.3]	28.5 [20.6, 42.4]	26.8 [20.2, 37.4]	25.4 [19.3, 34.2]	< 0.001

*BMI* body mass index; *COPD* chronic obstructive pulmonary disease; *LVEF* left ventricular ejection fraction; *NYHA* New York Heart Association; *eGFR* estimated glomerular filtration rate; *ACE/ARB* ACE-inhibitor/angiotensin receptor blocker; *MRA* mineralocorticoid receptor antagonist; *NT-proBNP* N-terminal pro B-type natriuretic peptide; *hs-cTnT* high-sensitive cardiac troponin T; *sST2* soluble suppression of tumorigenicity 2

Only a small proportion of patients had a BMI of ≤ 18.5 kg/m^2^ (*n* = 83, 1.4%). The majority of patients with BMI ≥ 30 kg/m^2^ had class 1 obesity (i.e. BMI 30.0–35.0 kg/m^2^; *n* = 905, 77.4%), 195 (16.7%) had class 2 (35.0–40.0 kg/m^2^) and 70 (6.0%) class 3 (> 40.0 kg/m^2^). As data did not differ in a clinically meaningful way between the three obesity groups, they were combined for group comparisons. Most measured patient characteristics differed significantly between the four groups based on BMI (Table 1). The only exceptions were the presence of atrial fibrillation and the percentage of receiving MRA’s. Patients with low BMI were older, less often male, differed regarding underlying cause of HF and the co-morbidity profile, had more severe symptoms and higher biomarker levels. When comparing patients aged below 75 years and no-comorbidities (*n* = 2049, 35%) with the other patients (*n* = 3770, 65%), significant differences were found regarding all baseline characteristics as shown in supplementary Table 1.

### Survival analysis

As previously shown [10], outcome (i.e. overall survival and survival free of HF hospitalization) in the lowest two BMI groups was significantly worse as compared to the other two groups (*p* < 0.001 for both analyses). Thus, overall 3-year survival was 57% and 68% in patients with BMI ≤ 18.5 kg/m^2^ and 18.5–25.0 kg/m^2^, respectively, as compared to 77% in the 25.0–30.0 kg/m^2^ and 78% in the > 30 kg/m^2^ BMI group. No differences were found between the three obesity groups. Three-year survival free of HF hospitalization was 42%, 50%, 58% and 56%, respectively, in the four BMI groups.

**Fig. 1:**
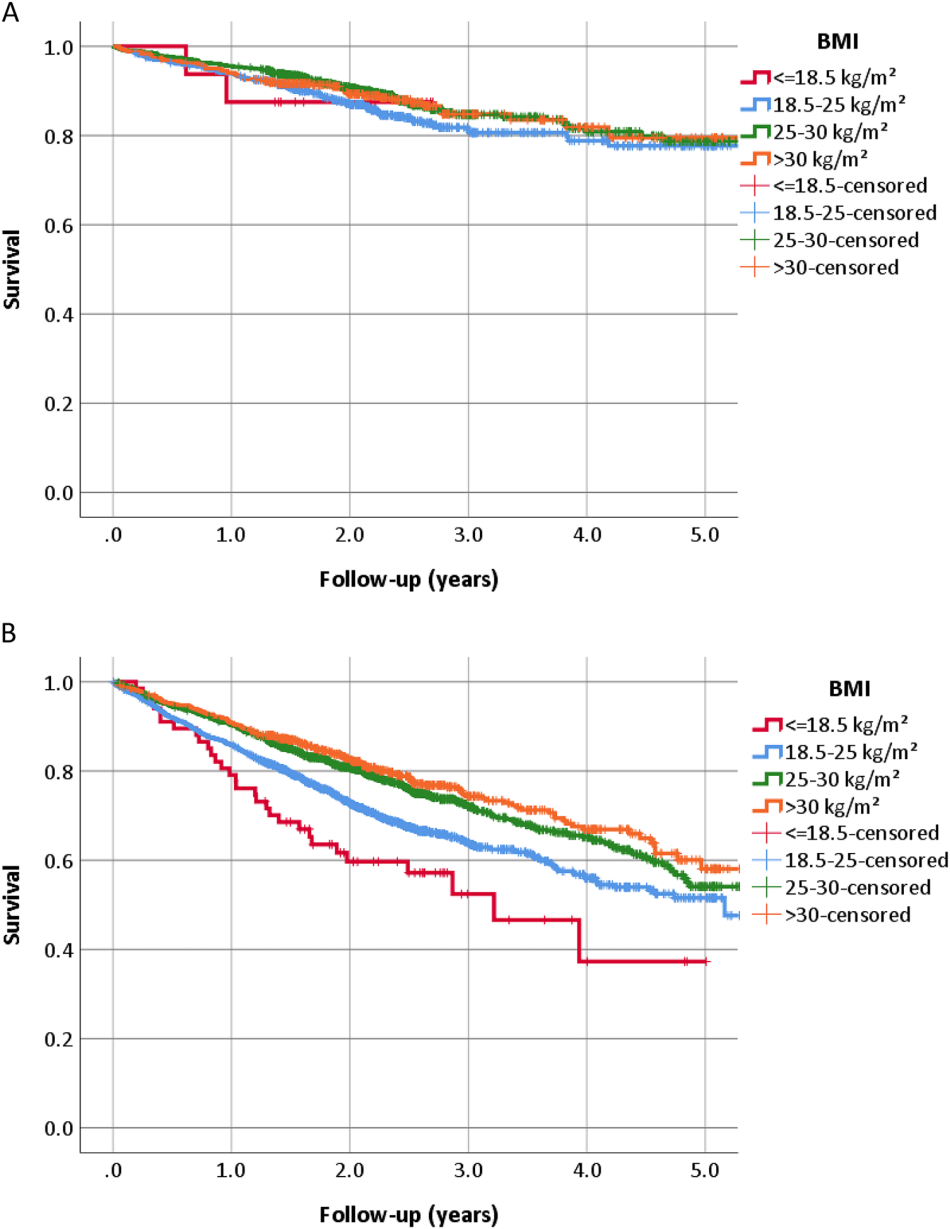
All-cause mortality in groups of body mass index (BMI) depending on the presence or absence of co-morbidities and age. **a** No co-morbidities and age < 75 years (*p* = 0.30); **b** at least one co-morbidity or age > 75 years (*p* < 0.001)

**Fig. 2:**
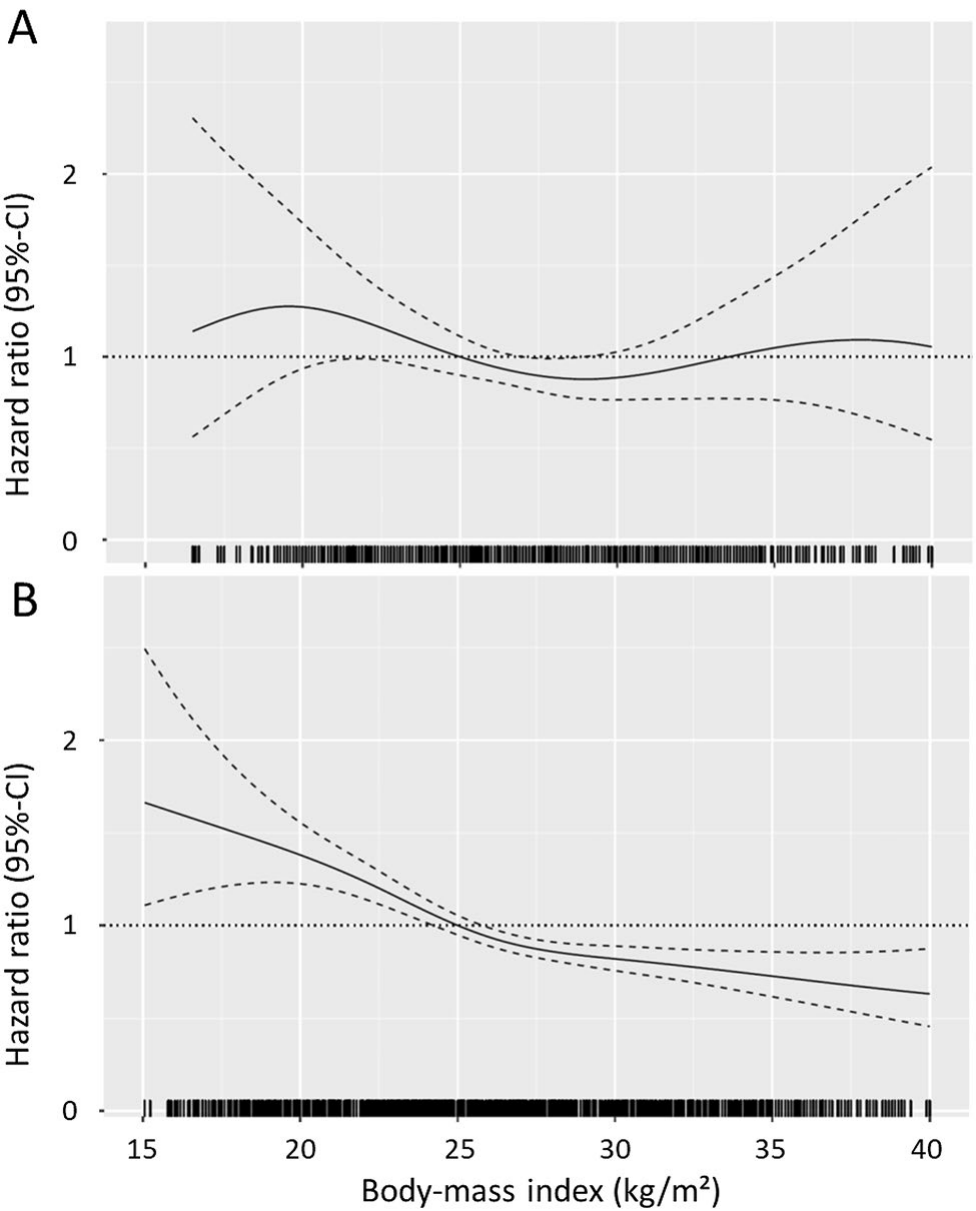
Body-mass index and risk of all-cause mortality: spline curve analysis. **a** No co-morbidities and age < 75 years; **b** At least one co-morbidity or age > 75 years. Due to very low numbers of patients truncated below 15 kg/m^2^ and above 40 kg/m^2^

When analysing patients aged below 75 years and no co-morbidities separately from the other patients, outcome was better in the former group and worse in the latter one. However, significant differences between the BMI groups were only present in patients with either co-morbidities or age of 75 years or more but not in those younger than 75 years of age and no co-morbidities (Figs. 1 and 2, supplementary Fig. 1, and supplementary Table 2). Outcome was largely similar in different obesity classes in both groups (Fig. 2).

### Multivariable predictors of outcome

In patients younger than 75 years without co-morbidities (anaemia, diabetes, COPD, renal failure, cancer), BMI in four categories was not significantly related to outcome (forced into equation). LVEF, severity of symptoms, ischaemic aetiology and eGFR were independent predictors of mortality. Regarding cardiovascular mortality, female gender was additionally related to less events (supplementary Table 3). Regarding survival free of HF hospitalization, atrial fibrillation and age replaced eGFR as predictor (Table 2).

**Table 2:** Multivariable Cox-regression to predict all-cause mortality excluding biomarkers

	Overall survival					Survival free of HF hospitalization				
	HR	95% CI		Wald	*p*	HR	95% CI		Wald	*p*
*A: No co-morbidities and age < 75 years*										
BMI categories				3.4	0.39				2.4	0.49
≤ 18.5 kg/m^2^	0.954	0.232	3.922	0.0	0.95	0.883	0.325	2.398	0.1	0.81
18.5–25.0 kg/m^2^	1.140	0.815	1.595	0.6	0.45	1.060	0.840	1.337	0.2	0.63
25.0–30.0 kg/m^2^	0.887	0.638	1.232	0.5	0.47	0.917	0.732	1.147	0.6	0.45
> 30.0 kg/m^2^	1				Ref	1				Ref
Age	–	–	–	–	–	1.010	1.001	1.019	5.1	0.02
eGFR	0.985	0.976	0.995	8.8	0.003	–	–	–	–	–
Ischaemic aetiology	1.562	1.219	2.002	12.1	< 0.001	1.267	1.069	1.501	7.4	0.004
NYHA III/IV	1.715	1.335	2.203	18.5	< 0.001	1.564	1.311	1.867	24.6	< 0.001
LVEF	0.960	0.944	0.976	25.0	< 0.001	0.975	0.965	0.986	20.0	< 0.001
Atrial fibrillation	–	–	–	–	–	1.389	1.104	1.748	7.9	0.005
*B: At least one co-morbidity or age > 75 years*										
BMI categories				22.4	< 0.001				10.3	0.02
≤ 18.5 kg/m^2^	2.037	1.366	3.039	12.2	< 0.001	1.320	0.927	1.880	2.4	0.12
18.5–25.0 kg/m^2^	1.214	1.009	1.461	4.2	0.04	1.115	0.969	1.285	2.3	0.13
25.0–30.0 kg/m^2^	0.963	0.802	1.156	0.2	0.69	0.951	0.829	1.090	0.5	0.47
> 30.0 kg/m^2^	1				Ref	1				Ref
Haemoglobin	0.911	0.874	0.949	20.1	< 0.001	0.911	0.882	0.941	31.9	< 0.001
Age	1.021	1.014	1.028	31.4	< 0.001	1.012	1.007	1.018	18.7	< 0.001
Cancer	1.223	1.016	1.473	4.5	0.03	–	–	–	–	–
COPD	1.266	1.098	1.459	10.6	0.001	1.374	1.228	1.538	30.8	< 0.001
eGFR	0.987	0.983	0.991	39.4	< 0.001	0.990	0.987	0.994	36.9	< 0.001
Ischaemic aetiology	1.232	1.080	1.406	9.6	0.002	1.158	1.045	1.283	7.9	0.005
Diabetes	1.194	1.050	1.357	7.3	0.007	1.318	1.192	1.457	29.1	< 0.001
Female gender	0.652	0.556	0.766	27.3	< 0.001	0.788	0.697	0.891	14.6	< 0.001
NYHA III/IV	1.527	1.347	1.731	43.7	< 0.001	1.340	1.215	1.478	34.4	< 0.001
Atrial fibrillation	1.206	1.035	1.404	5.8	0.02	1.250	1.106	1.413	12.7	< 0.001
LVEF	0.984	0.978	0.991	20.4	< 0.001	0.995	0.990	1.000	3.5	0.06

Abbreviations, see Table 1

In patients either older than 75 years or having at least one co-morbidity, BMI was an independent predictor of outcome, i.e. BMI ≤ 18.5 kg/m^2^ and 18.5–25.0 kg/m^2^ were accompanied by higher mortality. Still, the predictive value of cardiovascular mortality and survival free of HF hospitalization was less. Co-morbidities contributed to the risk of all outcomes, in addition to measures of severity of HF, age and male gender.

When including medication, NT-proBNP and hs-cTnT into the model, BMI was no longer significantly associated with outcome irrespective of the endpoint used and of the group investigated, apart from BMI < 18.5 kg/m^2^ having a higher mortality (Table 3). Both biomarkers, i.e. NT-proBNP and hs-cTnT, were very strong predictors of all outcome measures in both groups. The influence of other variables was somewhat less pronounced, but most of them remained significant predictors of outcome apart from BMI (Table 3, supplementary table 4). β-Blocker use was associated with better survival in those younger than 75 years without co-morbidities and ACE-inhibitor/ARB use with better survival free of HF hospitalization.

**Table 3:** Multivariable Cox-regression to predict all-cause mortality including biomarkers (apart from sST2) and medication

	Overall survival					Survival free of HF hospitalization				
	HR	95%-CI		Wald	*p*	HR	95% CI		Wald	*p*
*A: No co-morbidities and age < 75 years*										
BMI				1.9	0.60				1.9	0.55
≤ 18.5 kg/m^2^	0.821	0.198	3.408	0.1	0.79	0.881	0.323	2.402	0.1	0.81
18.5–25.0 kg/m^2^	1.025	0.727	1.446	0.0	0.89	1.047	0.826	1.329	0.1	0.70
25.0–30.0 kg/m^2^	0.860	0.618	1.197	0.8	0.37	0.921	0.735	1.154	0.5	0.47
> 30.0 kg/m^2^	1				Ref	1				Ref
Age						0.991	0.981	1.000	3.9	0.05
Ischaemic aetiology	1.724	1.348	2.205	18.8	< 0.001	1.357	1.146	1.608	12.5	< 0.001
NYHA III/IV	1.398	1.080	1.811	6.5	0.01	1.290	1.076	1.547	7.6	0.006
LVEF	0.972	0.956	0.988	11.4	0.001	0.987	0.976	0.998	5.4	0.02
Log NT-proBNP	1.772	1.307	2.403	13.6	< 0.001	1.653	1.344	2.033	22.7	< 0.001
Log hs-cTnT	2.505	1.764	3.559	26.3	< 0.001	2.730	2.150	3.467	67.8	< 0.001
RAS-blocker	–	–	–	–	–	0.591	0.457	0.765	16.0	< 0.001
β-Blocker	0.753	0.586	0.967	4.9	0.03	–	–	–	–	–
*B: At least one co-morbidity or age > 75 years*										
BMI				10.7	0.01				2.3	0.51
≤ 18.5 kg/m^2^	1.661	1.113	2.478	6.2	0.01	1.117	0.782	1.596	0.4	0.58
18.5–25.0 kg/m^2^	1.004	0.835	1.207	0.0	0.97	0.981	0.851	1.132	0.1	0.77
25.0–30.0 kg/m^2^	0.903	0.752	1.083	1.2	0.27	0.927	0.808	1.063	1.2	0.27
> 30.0 kg/m^2^	1				Ref	1				Ref
eGFR	0.995	0.991	0.999	7.3	0.007	0.996	0.993	0.999	6.5	0.01
Diabetes	–	–	–	–	–	1.175	1.062	1.300	9.8	0.002
Haemoglobin	–	–	–	–	–	0.955	0.925	0.987	7.8	0.005
COPD	–	–	–	–	–	1.281	1.143	1.434	18.3	< 0.001
Ischaemic aetiology	1.294	1.135	1.475	14.9	< 0.001	1.164	1.051	1.288	8.6	0.003
NYHA III/IV	1.276	1.123	1.450	14.1	< 0.001	1.171	1.060	1.294	9.7	0.002
Female gender	0.713	0.611	0.832	18.4	< 0.001	0.872	0.771	0.986	4.8	0.03
Log NT-proBNP	2.198	1.884	2.566	99.8	< 0.001	1.516	1.348	1.704	48.6	< 0.001
Log hs-cTnT	2.056	1.730	2.442	67.3	< 0.001	2.081	1.815	2.387	109.9	< 0.001
RAS-blocker	–	–	–	–	–	0.861	0.747	0.992	4.3	0.04

Abbreviations, see Table 1

### Factors influencing biomarker levels

**Fig. 3:**
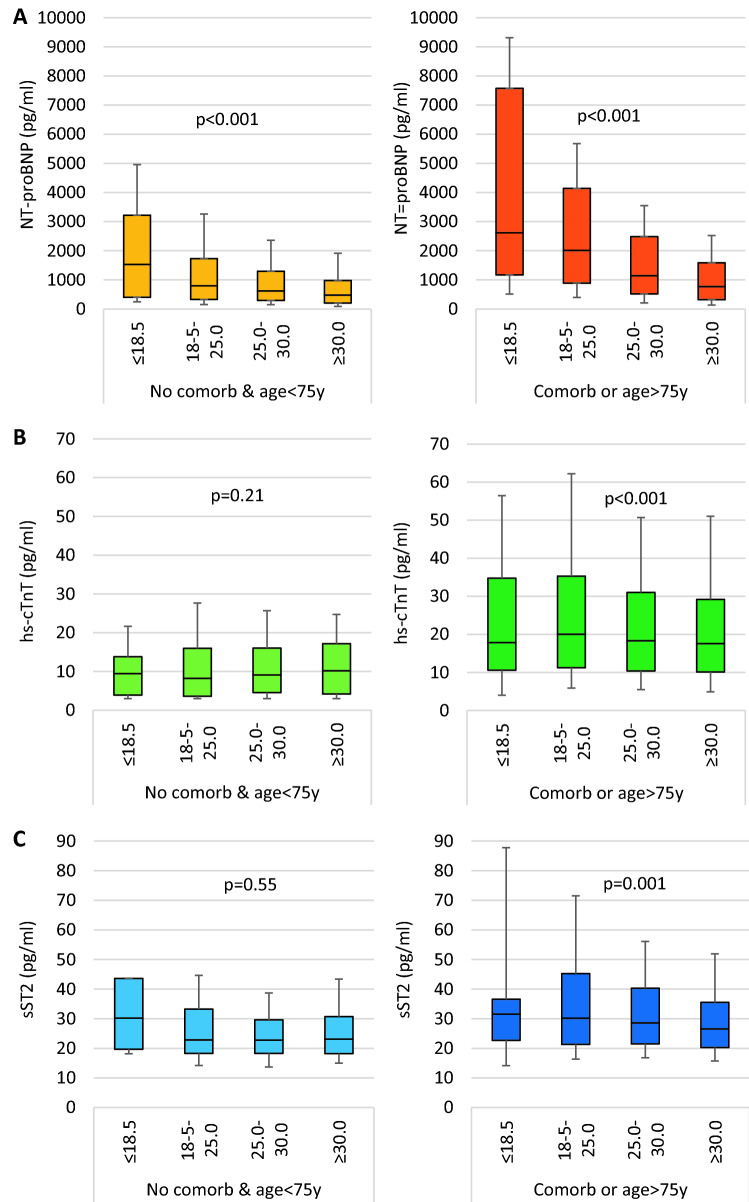
Biomarker levels in patients without co-morbidities and aged below 75 years as compared to patients with at least one co-morbidity or aged above 75 years. **a** NT-proBNP, **b** hs-cTnT, **c** sST2

Levels of all biomarkers were significantly lower in patients aged < 75 years without non-cardiovascular co-morbidities as compared to the other patients (NT-proBNP 646 [IQR 287–1360] versus 1314 [555–2948] pg/ml, hs-cTnT 8.9 [4.1–16.2] versus 18.7 [10.6–32-5] pg/ml, sST2 22.9 [18.3–30.5] versus 28.7 [21.1–40.8] ng/ml, respectively, all *p* < 0.001). NT-proBNP levels were inversely related to BMI groups in both patient groups, i.e. aged < 75 years without co-morbidities versus all other patients (Fig. 3a). In contrast, this association was less for hs-cTnT and sST2 and only seen in patients aged > 75 years and/or at least one co-morbidity, whereas no association with BMI was present in those aged < 75 years and no co-morbidities (Fig. 3b,c).

In multivariable linear regression analysis, various factors were associated with biomarker levels (supplementary table 5). NT-proBNP levels were inversely correlated with BMI, whereas the association with BMI was minimal for sST2 and absent for hs-cTnT. Age was positively correlated with all biomarkers, particularly regarding NT-proBNP and hs-cTnT levels. Severity of symptoms and the presence of atrial fibrillation were independently correlated with higher levels of all biomarkers. Higher LVEF was associated with lower NT-proBNP and hs-cTnT levels. Co-morbidities influenced levels of biomarkers and effects were highest for hs-cTnT. Finally, MRA use was associated with higher biomarker levels, whereas β-blockers were associated with higher NT-proBNP and sST2 but lower hs-cTnT levels. The effects were not different in a meaningful way when separating the groups into younger patients and no-co-morbidities and the other patients (supplementary table 6).

## Discussion

The present analysis provides several new aspects regarding the importance of BMI in patients with chronic HF, particularly with reduced LVEF (HFrEF). First, the association between lower BMI and worse outcome may be largely explained by the combination of HF severity and co-morbidities, suggesting lower body weight is mainly a sign of more advanced disease rather than a causative factor of poor outcome. These results are consistent with previous data in those with acute HF [11]. Second, there was no obesity paradox in HF patients that did not have comorbidities and were relatively young, further supporting that normal body weight is not contributing to increased risk nor is obesity protective in HF. Third, the influence of BMI on NT-proBNP levels is independent of other factors including HF severity and co-morbidities, but basically absent for hs-cTnT and sST2. Still, all three markers are significantly influenced by multiple factors other than cardiac.

### The “obesity paradox” in perspective

Numerous studies have found generally lower risk in overweight and obese patients with HF, as compared to patients with normal body weight [5, 10] and nicely summarised in a recent meta-analysis [12], unless obesity is extreme [13]. Some exceptions have been described based on HF aetiology (ischaemic vs. non-ischaemic) [14, 15] or the presence of diabetes [16]. Physiology in HF may change, and factors that increase the risk of HF such as obesity might become unimportant or even protective. This may explain why results on the prognostic impact of obesity differ between studies [12]. Reverse epidemiology has been described not only for obesity but also for cholesterol levels and hypertension [17]. Such reverse epidemiology might also explain the lack of effect by statin treatment in HFrEF, even if the underlying cause is coronary artery disease [18]. There are several potential mechanistic explanations for this, based on some but not yet uniform and/or convincing evidence. Thus, various anti-inflammatory adipokines may have beneficial effects in HF [5]. They include, e.g. soluble tumour necrosis factor-α receptor, which may neutralize some components of inflammation. The higher lipoprotein levels may counteract circulating inflammatory endotoxin seen in HF [19]. In addition, adiponectin levels are lower in obesity and increased levels can increase energy expenditure and induce weight loss, which is undesirable in the catabolic state of HF [17]. Finally, a mild elevation in circulating progenitor cells has been found in healthy individuals with obesity [20]. Even though related to future metabolic deterioration, it may be hypothesized that these cells might be beneficial in areas of myocardial injury in patients with HF and potentially explaining the divergent effect in HF patients as compared to healthy individuals.

There may also be explanations for the obesity paradox that are not directly related to beneficial effects of obesity per se. Thus, obese patients may become symptomatic at an earlier stage of HF than lean subjects, resulting in less advanced HF at the time of diagnosis and earlier implementation of life-saving therapy. Obese HF patients typically have higher blood pressure despite attenuated response to the renin–angiotensin–aldosterone system, leaving more room to establish optimal guideline-recommended medical therapy, which may improve outcome at least in patients with HFrEF [21]. Moreover, the majority of studies measures obesity by body mass index, but studies utilizing less-frequently used measures of body fat and body composition, including waist circumference, waist–hip ratio, skinfold estimates, and bioelectrical impedance analysis also confirmed the obesity paradox in HF [22]. Thus, alternative explanations such as higher BMI caused by increase in muscle mass and increased fitness in some patients [23], BMI not representing body fat with significant variation depending on sex, age and ethnicity [24] or variation in levels of visceral adipose tissue [25] are unlikely to solve the obesity paradox.

Lastly, overweight and obesity may signify enough reserve while patients at lower body may have more advanced HF and comorbidities, some of whom may suffer from unintentional weight loss, i.e. cardiac cachexia. In fact, cachexia is not only related to worse outcome, but can also be prevented by treatment of HF [26]. The results of the present study suggest that the presence of co-morbidities and the severity of HF indeed may explain the obesity paradox to a large extent. This was the hypothesis of this analysis and the reason for investigating a ‘pure’ HF group with little or no influencing factors related to BMI. Indeed in younger patients with no relevant co-morbidities, no significant prognostic effect of BMI was found anymore, even in univariable analysis. Moreover, in a fully adjusted model including biomarkers representing the severity of HF and co-morbidities, the prognostic effect of BMI disappeared, apart from mortality in those with very low BMI (i.e. < 18.5 kg/m^2^) suggesting at least in some of these patients cachexia. Findings of a prognostic impact of obesity only in patients with low peak oxygen uptake [27] or low fitness level [28] are in line with the present results as they suggest cardiac cachexia being the main driver for poor outcome.

### Cardiac biomarkers and obesity

The strong and independent influence of BMI on NT-proBNP levels in contrast to hs-cTnT and sST2 has been reported previously [10]. We extend on these findings and show that this association is not influenced by age and the presence of co-morbidities although these factors also impact NT-proBNP levels. The pathophysiology behind the decreasing levels of natriuretic peptides with increasing obesity is not yet fully understood. Several studies investigating not only BMI but also body fat percentage [10], fatty liver disease [29], glucose tolerance and insulin resistance [30, 31] in HF and non-HF patients, found similar correlations and suppose the amount of adipose tissue to be an important contributing factor in decreasing NT-proBNP levels. Moreover, if adipose tissue is lowered after lifestyle intervention, NT-proBNP levels increase [32]. Adipose tissue is a source of sex hormones, which have been liked to suppression of NT-proBNP secretion; in analyses fitting concentrations of androgens to linear regression models, association between BMI and NT-proBNP was muted [33]. Alterations in metabolism of pro-BNP into different fragments and glycosylation that may not be adequately detected by current assays [34] but also different expression of natriuretic peptide clearance receptors [31] are other potential explanations of the finding. On the other hand, the fact that natriuretic peptides are influenced by multiple other factors in addition to age independently of cardiac function is often ignored although they are known for a long time already [35]. The present results suggest that such an influence is also present in chronic HF, which needs to be considered when interpreting natriuretic peptide levels.

Interestingly, hs-cTnT levels were significantly influenced not only by cardiac factors including markers of the severity of HF but also independently by most of the evaluated co-morbidities whereas the impact of BMI was minor. The exact reason for non-cardiac elevation of cardiac troponins is not well known. The purely cardiac origin of them makes (an indirect) cardiac involvement likely. In addition, metabolism may be altered. Still, alternative mechanisms might be considered such as alteration in turnover of cardiac troponins. Different types of troponins may not be affected similarly as shown in elderly comorbid patients [36]. Finally, both the cardiac and the non-cardiac influence on sST2-levels were smaller than for the other two biomarkers, despite a strong impact on outcome across the whole range of BMI [10] and independent of LVEF [37].

### Limitations

Some limitations apply to this analysis. First, direct measures of body composition are not available. Second, the number of co-morbidities collected is limited and was not harmonized between the included studies. No information on the severity of co-morbidities (apart from the measurement of eGFR) was available. In addition, information was lacking in some studies. Therefore, information on co-morbidities is not complete. Third, patients were enrolled in trials, making it likely that a certain selection bias was applied. The average age and the percentage of women, both of which are lower than in the general HF population, are expressions of this. Therefore, patients with more advanced age and likely more prevalent co-morbidities were not included in the studies. Fourth, no information on the nutritional status and changes in body weight over time is available. Therefore, a distinction between (cardiac) cachexia and stable normal body weight with normal nutritional and metabolic state is not possible. Fifth, the information on cardiac function and severity of HF is limited. Also, no information is available on muscle structure and function [38] or exercise tolerance, which may affect the relationship between BMI and outcome [39]. Despite these limitations, a clear impact of the variables investigated could be shown making it unlikely that the present results are chance findings only.

## Conclusions

The data of the present study call a direct protective role of obesity in HFrEF into question and suggest that obesity is merely a marker of better prognosis due to less advanced HF and less co-morbidities. This does not mean that losing weight in obese HF patients may result in better outcome. In fact, there may be a rather neutral effect of obesity, unless patients are extremely obese (BMI > 40 kg/m^2^). Still, prospective intervention trials are required to clearly answer the question of the exact pathophysiological role of obesity in chronic HF.

## Availability of data and material

Original data may be requested from the corresponding author after signing a confidentiality agreement and after providing the purpose of this request.

## Supplementary Information

Below is the link to the electronic supplementary material.

Supplementary file1 (PDF 116 KB)

Supplementary file2 (DOCX 59 KB)

Supplementary file3 (DOCX 27 KB)

## Data Availability

Not applicable.
