# Supplementary figures and images for: Re-appraisal of the obesity paradox in heart failure: a meta-analysis of individual data
Source: Clin Res Cardiol. 2021 Mar 11;110(8):1280–91. doi: 10.1007/s00392-021-01822-1 (PMC8318940; doi:10.1007/s00392-021-01822-1)

A

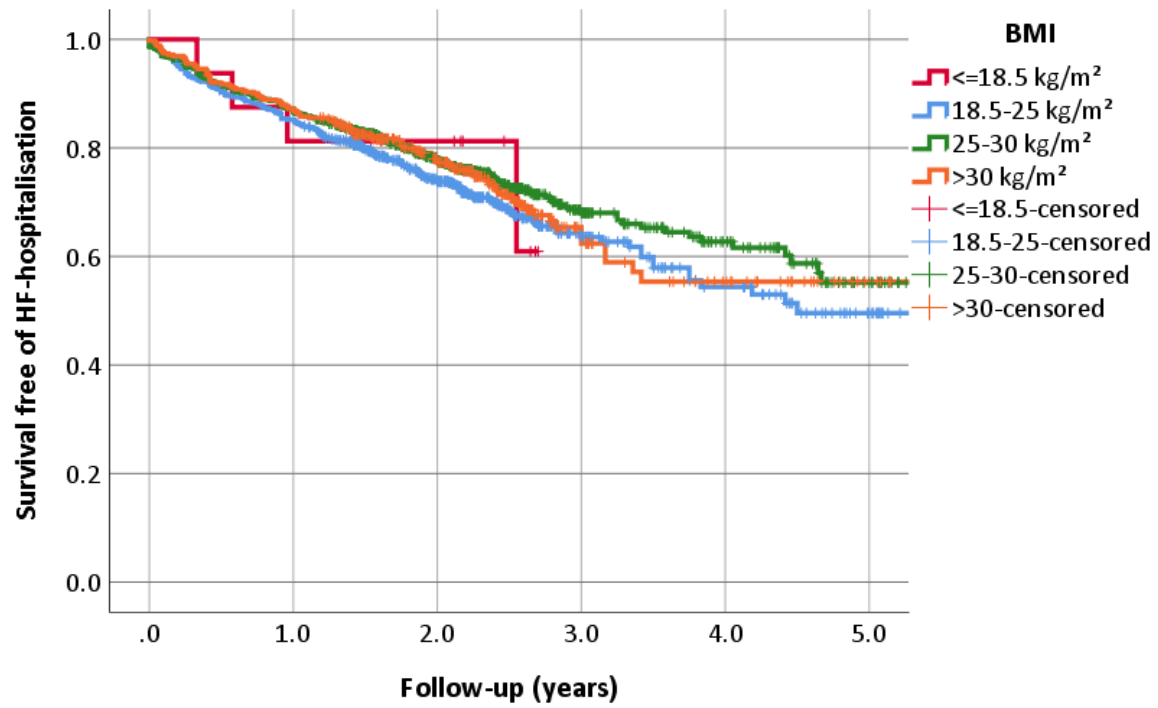

B

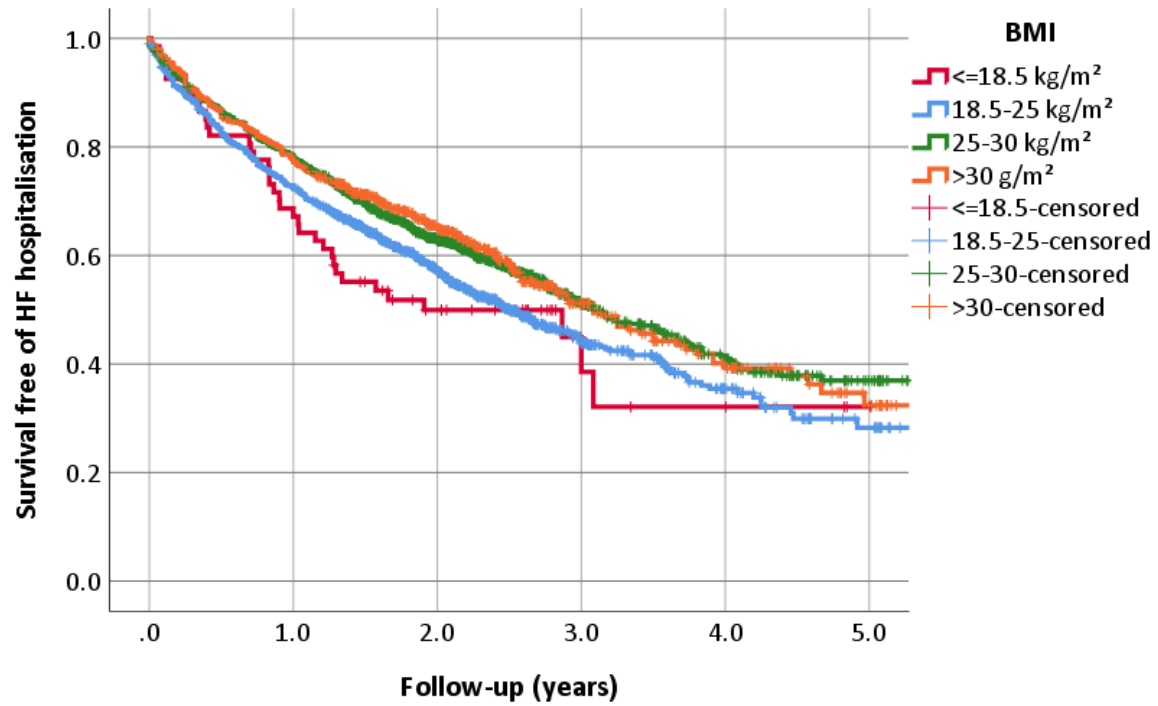

Supplement: Supplementary file 1 — Supplementary file1 (PDF 116 KB) [file 392_2021_1822_MOESM1_ESM.pdf]
