# Supplementary material for: Re-appraisal of the obesity paradox in heart failure: a meta-analysis of individual data
Source: Clin Res Cardiol. 2021 Mar 11;110(8):1280–91. doi: 10.1007/s00392-021-01822-1 (PMC8318940; doi:10.1007/s00392-021-01822-1)

**Data Sources, Search Strategy and Eligibility Criteria**

The meta-analysis was conducted in agreement with the Preferred Reporting Items for Systematic reviews and Meta-Analyses statement[1]. In April 2017, 2 authors (Alberto Aimo, Giuseppe Vergaro) independently searched 4 databases (Medline, EMBASE, Cochrane Library, and Scopus). The search terms, deliberately broad, were “troponin” AND “heart failure” OR “cardiac failure” OR “cardiac dysfunction” OR “cardiac insufficiency” OR “left ventricular dysfunction.”

Inclusion criteria were as follows:

1. English language

2. Patients aged ≥18 years and diagnosed with HF *(clinical diagnosis based on contemporary guidelines)*

3. Reported enrollment of outpatients or patients undergoing elective admission

4. Reported use of a hs assay for TnT and TnI

5. Information on patient prognosis

6. Authors’ availability to provide individual patient data (IPD).

*References exported to Excel*

Two authors (A.A., G.V.) independently evaluated the studies to be included in the meta-analysis. Disagreement about study inclusion was resolved by discussion with a third author (M.E.).

The following data were extracted from each study: first author’s last name, publication year, country where the study was conducted, study period, number of patients enrolled, left ventricular ejection fraction (LVEF) criteria, follow-up duration, outcomes evaluated, and all demographic and clinical baseline data provided. The data were extracted by a reviewer (A.A.), and checked for accuracy by a second reviewer (G.V.). The authors of each study were then contacted and asked to provide IPD for as many as possible of the following variables: age, sex, ethnic group (black versus others), body mass index, hypertension, atrial fibrillation, diabetes mellitus, chronic obstructive pulmonary disease, plasma hemoglobin, HF etiology (ischemic versus nonischemic), LVEF, hs-TnT and hs-TnI, natriuretic peptides (NPs: either B-type NP [BNP] or the N-terminal fraction of its precursor [NT-proBNP]), serum creatinine, hs C-reactive protein, follow-up duration, and outcome measures (all-cause death, cardiovascular death, and hospitalization for cardiovascular cause). As hs-TnI values were available for a small minority of patients, only hs-TnT was considered in analysis. The selection steps are shown in the figure below. The list of included studies can be found in the first publication of this meta-analysis based on individual patient data[2]. This analysis was not sponsored, and there were no sources of funding.

Of the 9,283 patients selected, data on BMI, co-morbidities and outcome was available in 5,819 patients. They formed the basis for the present analysis.

**Reference**

1. Moher D, Liberati A, Tetzlaff J, Altman DG (2009) Preferred reporting items for systematic reviews and meta-analyses: the PRISMA statement. PLoS medicine 6 (7):e1000097. doi:10.1371/journal.pmed.1000097

2. Aimo A, Januzzi JL, Jr., Vergaro G, Ripoli A, Latini R, Masson S, Magnoli M, Anand IS, Cohn JN, Tavazzi L, Tognoni G, Gravning J, Ueland T, Nymo SH, Brunner-La Rocca HP, Bayes-Genis A, Lupon J, de Boer RA, Yoshihisa A, Takeishi Y, Egstrup M, Gustafsson I, Gaggin HK, Eggers KM, Huber K, Tentzeris I, Tang WHW, Grodin J, Passino C, Emdin M (2018) Prognostic Value of High-Sensitivity Troponin T in Chronic Heart Failure: An Individual Patient Data Meta-Analysis. Circulation 137 (3):286-297. doi:10.1161/circulationaha.117.031560


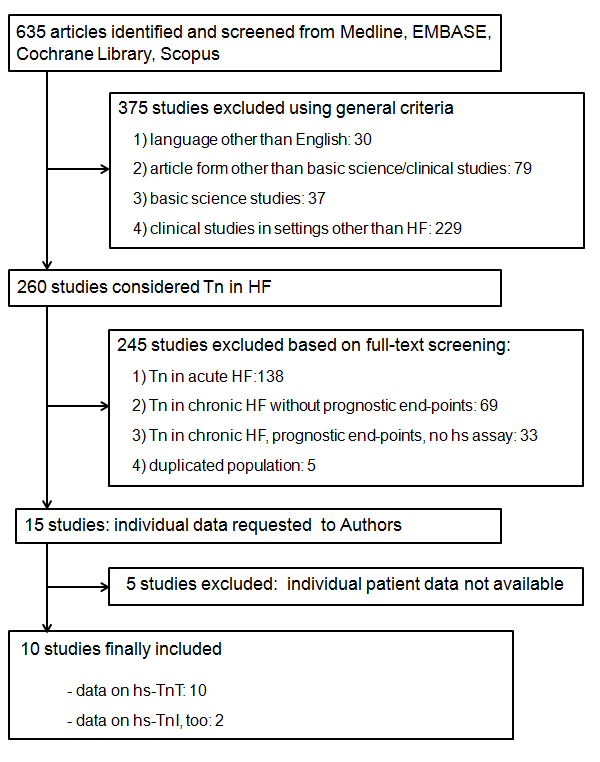

Supplement: Supplementary file 2 — Supplementary file2 (DOCX 59 KB) [file 392_2021_1822_MOESM2_ESM.docx]
