# Supplementary material for: Re-appraisal of the obesity paradox in heart failure: a meta-analysis of individual data
Source: Clin Res Cardiol. 2021 Mar 11;110(8):1280–91. doi: 10.1007/s00392-021-01822-1 (PMC8318940; doi:10.1007/s00392-021-01822-1)
Supplement: Supplementary file 3 — Supplementary file3 (DOCX 27 KB) [file 392_2021_1822_MOESM3_ESM.docx]

Supplementary table 1 Baseline characteristics in patients aged <75 years and no-comorbidities as compared to the rest of the patients

|  | All (n=5,819) | No co-morbidities and age <75 years (n=2,049) | At least one co-morbidity or age >75 years (n=3,770) | P |
| --- | --- | --- | --- | --- |
| Age (years) | 64.9±11.5 | 59.0±10.3 | 68.2±10.8 | <0.001 |
| Male gender | 4561 (78.4%) | 1721 (84.0%) | 2840 (75.3%) | <0.001 |
| BMI (kg/m²) | 26.8±4.5 | 27.1±4.2 | 26.7±4.6 | 0.002 |
| Ischemic etiology | 3256 (56.0%) | 1010 (49.3%) | 2246 (59.6%) | <0.001 |
| Hypertension | 2776 (47.7%) | 751 (36.7%) | 2025 (53.7%) | <0.001 |
| Cancer | 427 (7.3%) | - | 427 (11.3%) | n.a. |
| COPD | 860 (14.8%) | - | 860 (22.8%) | n.a. |
| Diabetes | 1563 (26.9%) | - | 1563 (41.5%) | n.a. |
| Hemoglobin (g/dl) | 13.6±1.5 | 14.4±1.1 | 13.2±1.6 | <0.001 |
| Anemia | 1605 (27.6%) | - | 1605 (42.6%) | n.a. |
| eGFR | 58.8±17.2 | 65.8±14.0 | 55.0±17.5 | <0.001 |
| eGFR<45 | 1192 (20.5%) | - | 1192 (31.6%) | n.a. |
| Atrial fibrillation | 893 (15.4%) | 261 (12.8%) | 632 (16.8%) | <0.001 |
| LVEF | 28.9±9.1 | 28.2±7.7 | 29.3±9.7 | <0.001 |
| NYHA III/IV | 2282 (39.2%) | 574 (28.0%) | 1708 (45.3%) | <0.001 |
| ACE/ARB | 5242 (90.1%) | 1885 (92.0%) | 3357 (89.1%) | <0.001 |
| β-blocker | 2706 (46.5%) | 998 (48.7%) | 1708 (45.3%) | 0.013 |
| MRA | 954 (16.4%) | 242 (11.8%) | 712 (18.9%) | <0.001 |
| NT-proBNP (pg/ml) | 1001  [416, 2345] | 646 [287, 1360] | 1314 [555, 2948] | <0.001 |
| hs-TnT (pg/ml) | 14.8  [7.5, 26.3] | 8.9 [4.1, 16.2] | 18.7 [10.6, 32.5] | 0.001 |
| sST2 (ng/ml) | 26.9  [20.2, 38.5] | 22.9 [18.3, 30.5] | 28.7 [21.1, 40.8] | <0.001 |

Abbreviations: BMI body mass index; LVEF left-ventricular ejection fraction; NYHA New York Heart Association; ACE/ARB ACE-inhibitor/angiotensin receptor blocker; MRA mineralocorticoid receptor antagonist; n.a. not applicable

Supplementary table 2 Multivariable predictors of biomarker levels in linear regression analysis

|  | NTpro-BNP (log 10)  Adjusted R² = 0.36 | | hs-TnT (log 10)  Adjusted R² = 0.36 | | sST2 (log 10)  Adjusted R² = 0.17 | |
| --- | --- | --- | --- | --- | --- | --- |
|  | B | p | B | p | B | P |
| Age | 0.012 | <0.001 | 0.011 | <0.001 | 0.003 | <0.001 |
| Female gender | - | - | -0.186 | <0.001 | -0.085 | <0.001 |
| BMI | -0.023 | <0.001 | - | - | -0.002 | 0.02 |
| eGFR | -0.005 | <0.001 | -0.004 | <0.001 | -0.001 | <0.001 |
| Diabetes | 0.035 | 0.01 | 0.148 | <0.001 | 0.040 | <0.001 |
| COPD | - | - | 0.086 | <0.001 | - | - |
| Hypertension | 0.058 | <0.001 | 0.092 | <0.001 | - | - |
| Haemoglobin | -0.043 | <0.001 | -0.031 | <0.001 | -0.007 | 0.04 |
| Ischaemic aetiology | - | - | -0.039 | <0.001 | - | - |
| LVEF | -0.013 | <0.001 | -0.004 | <0.001 | - | - |
| NYHA III/IV | 0.178 | <0.001 | 0.107 | <0.001 | 0.064 | <0.001 |
| Atrial fibrillation | 0.261 | <0.001 | 0.075 | <0.001 | 0.086 | <0.001 |
| MRA | 0.098 | <0.001 | 0.119 | <0.001 | 0.069 | <0.001 |
| β-blocker | 0.053 | <0.001 | -0.042 | 0.002 | 0.019 | 0.04 |

Supplementary table 3 Multivariable predictors of biomarker levels in linear regression analysis in patients separated based on age below and above 75 years and co-morbidities

A: No comorbidities, age <75 years

|  | NTpro-BNP (log 10) | | hs-TnT (log 10) | | sST2 (log 10) | |
| --- | --- | --- | --- | --- | --- | --- |
|  | B | p | B | p | B | p |
| Age | 0.012 | <0.001 | 0.010 | <0.001 | 0.002 | 0.006 |
| Female gender | - | - | -0.162 | <0.001 | -0.088 | <0.001 |
| BMI | -0.020 | <0.001 | 0.004 | 0.01 | - | - |
| eGFR | -0.002 | 0.004 | -0.001 | 0.03 | - | - |
| Hypertension | 0.044 | 0.03 | 0.097 | <0.001 | - | - |
| Ischaemic aetiology | -0.043 | 0.03 | -0.043 | 0.005 | 0.034 | 0.03 |
| LVEF | -0.018 | <0.001 | -0.007 | <0.001 | - | - |
| NYHA III/IV | 0.164 | <0.001 | 0.106 | <0.001 | 0.047 | <0.001 |
| Atrial fibrillation | 0.309 | <0.001 | 0.079 | <0.001 | 0.101 | <0.001 |
| MRA | 0.133 | <0.001 | 0.168 | <0.001 | 0.085 | 0.02 |
| BB | - | - | -0.056 | <0.001 | - | - |

B: At least one comorbidity or age >75 years

|  | NTpro-BNP (log 10) | | hs-TnT (log 10) | | sST2 (log 10) | |
| --- | --- | --- | --- | --- | --- | --- |
|  | B | p | B | p | B | P |
| Age | 0.012 | <0.001 | 0.011 | <0.001 | 0.003 | <0.001 |
| Female gender |  |  | -0.188 | <0.001 | -0.074 | <0.001 |
| BMI | -0.024 | <0.001 | - | - | -0.003 | 0.01 |
| eGFR | -0.005 | <0.001 | -0.005 | <0.001 | -0.001 | <0.001 |
| Diabetes | - | - | 0.131 | <0.001 | 0.035 | 0.002 |
| COPD | - | - | 0.073 | <0.001 | - | - |
| Cancer | - | - | - | - | -0.031 | 0.03 |
| Hypertension | 0.060 | <0.001 | 0.080 | <0.001 | - | - |
| Haemoglobin | -0.042 | <0.001 | -0.033 | <0.001 | - | - |
| Ischaemic aetiology | - | - | -0.032 | 0.007 | - | - |
| LVEF | -0.011 | <0.001 | -0.003 | <0.001 | - | - |
| NYHA III/IV | 0.178 | <0.001 | 0.102 | <0.001 | 0.067 | <0.001 |
| Atrial fibrillation | 0.227 | <0.001 | 0.069 | <0.001 | 0.085 | <0.001 |
| MRA | 0.077 | <0.001 | 0.094 | <0.001 | 0.071 | <0.001 |
| β-blocker | 0.070 | <0.001 | -0.035 | 0.002 | - | - |
